# Posttraining Outcomes, Acceptability, and Technology-Based Delivery of the STAC Bystander Bullying Intervention Teacher Module: Mixed Methods Study

**DOI:** 10.2196/40022

**Published:** 2022-08-03

**Authors:** Aida Midgett, Diana M Doumas, Mary K Buller

**Affiliations:** 1 Department of Counselor Education Boise State University Boise, ID United States; 2 Institute for the Study of Behavioral Health and Addiction Boise State University Boise, ID United States; 3 Klein Buendel, Inc Golden, CO United States

**Keywords:** teacher bullying interventions, technology-based bullying intervention, STAC, middle school

## Abstract

**Background:**

Bullying is a significant problem for youth associated with wide-ranging negative consequences. Providing students who witness bullying with intervention strategies to act as *defenders* can reduce bullying and negative associated outcomes for both targets and bystanders. Educating teachers about bullying and training them to support students to intervene as *defenders* may increase the efficacy of bystander programs as teachers’ attitudes and responses to bullying relate to bystander behavior. This is particularly important in middle school, when bullying peaks and rates of reporting bullying to teachers begin to decline. Reducing implementation barriers, including limited time and resources, must also be considered, particularly for schools in low-income and rural areas. Technology-based programs can increase access and scalability but require participant buy-in for adoption.

**Objective:**

We used a mixed methods design to inform the development of the STAC teacher module, a companion training to a brief bullying bystander intervention. STAC stands for the four bystander intervention strategies: *Stealing the Show*, *Turning it Over*, *Accompanying Others*, and *Coaching Compassion*. Objectives included examining the effectiveness of the STAC teacher module and informing the translation of the training into a technology-based format that can be used as a companion to the technology-based STAC.

**Methods:**

A sample of 17 teachers recruited from 1 middle school in a rural, low-income community completed pre- and posttraining surveys assessing immediate outcomes (ie, knowledge, confidence, comfort, and self-efficacy), intention to use program strategies, and program acceptability and relevance, followed by a qualitative focus group obtaining feedback regarding program appropriateness, feasibility, content, perception of need, and desire for web-based training. Descriptive statistics, 2-tailed independent-sample *t* tests, and thematic analyses were used to analyze the data.

**Results:**

Assessment of pre- and posttraining surveys indicated that teachers reported an increase in knowledge and confidence to support *defenders*, confidence and comfort in managing bullying, and bullying self-efficacy. Furthermore, most participants reported that they were likely or very likely to use STAC strategies to support students who intervene in bullying. Quantitative and qualitative data revealed that participants found the training easy to use, useful, relevant, and appropriate. Qualitative data provided feedback on ways of improving the program, including revising role-plays and guidance on understanding student behavior. Participants shared positive perceptions regarding program feasibility and need for bullying-specific prevention, the most significant barriers being cost and parent buy-in, suggesting the importance of including parents in the prevention process. Finally, participants shared the strengths of a web-based program, including ease of implementation and time efficiency, while indicating the importance of participant engagement and administration buy-in.

**Conclusions:**

This study demonstrates the effectiveness of the STAC teacher module in increasing knowledge and bullying self-efficacy and provides support for developing the module, including key information regarding considerations for web-based translation.

## Introduction

### Background

Bullying is a significant problem for youth, which is associated with a wide range of socioemotional consequences in childhood and adolescence [1] that extend into adulthood [2]. Bullying peaks in middle school, with 28% and 33% of students in the United States reporting being bullied at school and cyberbullied, respectively [3]. Among middle school students, being a target of bullying is associated with poor academic performance [4,5], absenteeism [5,6], somatic symptoms [7], anxiety [1,7], depression [1,7,8], suicidal ideation, and suicide attempts [1]. A growing body of literature demonstrates that students who witness school bullying as bystanders also report negative outcomes, including an increased risk of depression, anxiety [9-11], and somatic symptoms [12]. Similar trends are also emerging for middle school students who witness cyberbullying [13-15].

### Bystanders as Defenders

Most students (ie, up to 80%) report witnessing school bullying as bystanders [11], and approximately 50% report witnessing cyberbullying [16]. However, only 20% to 30% of students report intervening in school bullying [17], and as few as 10% report intervening in cyberbullying [18]. When bystanders interrupt a bullying situation by telling the bully to stop or by reporting bullying to an adult, bullying decreases [19] and bystanders report improved emotional adjustment [20]. However, often, bystanders do not intervene as they lack the knowledge or skills to act as *defenders* [21,22]. Therefore, equipping bystanders with strategies that they can use when they witness bullying can reduce both bullying and the negative associated consequences for both targets and students who witness bullying.

### The STAC Intervention

Researchers developed the STAC intervention [23] to train middle school students to act as *defenders* on behalf of targets of bullying using the following four intervention strategies: (1) *Stealing the Show* (using humor or distraction to interrupt a bullying situation and remove the attention from the target), (2) *Turning it Over* (identifying a trusted adult at school, reporting, and asking for help during a bullying incident), (3) *Accompanying Others* (befriending or providing support to a peer who was a target of bullying), and (4) *Coaching Compassion* (gently confronting the perpetrator and increasing empathy for the target). STAC is a brief bystander intervention comprising a 75-minute training that includes didactic and experiential components. In the didactic portion, students learn about the definition of bullying, types of bullying, negative associated consequences, bystander roles, and the 4 STAC strategies. The experiential component comprises role-plays during which students practice using the STAC strategies and receive feedback from the trainers. After the training, students participate in two 15-minute booster sessions to reinforce learning and skills. Boosters include check-in, support, and brainstorming of how to use the STAC strategies more effectively.

Research conducted with middle school students has shown that students report posttraining increases in knowledge of bullying and the STAC strategies, as well as the confidence to intervene on behalf of targets [23-26]. The findings also indicate that the STAC intervention is effective in reducing bullying victimization [24,27] and perpetration [24], as well as improving bystander mental health, including reducing anxiety [24,28], depression [24,29], and suicidal ideation [29], and increasing self-esteem [30].

### Use of STAC Strategies to Act as a Defender

Although ≥90% of middle school students report using at least one of the four STAC strategies to intervene in bullying situations after training [24,26,31], middle school students report using *Turning it Over* less frequently than elementary school students. Specifically, the prevalence rates of using “Turning it Over” declined from 78% in elementary school [31] to 69.1% in middle school [26]. Interestingly, this is not the case for the other 3 strategies, with prevalence rates increasing from 50.9% to 69.1% for *Stealing the Show*, from 76.4% to 90% for *Accompanying Others*, and from 44.4% to 69.1% for *Coaching Compassion* among elementary school students [31] and middle school students [26], respectively. A possible explanation for the lower rates of using “Turning it Over” in middle school is that middle school students may believe that teachers do not care enough about bullying to take action and perceive bullying as a less significant problem than students do [25]. Therefore, providing education to teachers about bullying and training them on how to support students when they report bullying or ask for help may be important additions to the STAC intervention at the middle school level.

### Equipping Teachers to Support Student Defenders

Middle school students indicate that school bullying occurs most often in the classroom, accounting for 42.3% of all school bullying incidences [32]. However, national data indicate that only approximately half (51.7%) of middle school students report bullying to an adult at school, with report prevalence declining from sixth grade (57.2%) to eighth grade (47%) [32]. Although students report bullying to teachers more often than to any other adult at school [33], reporting behavior is influenced by a student’s perception of teachers’ responses to bullying. Students are more likely to report when they believe teachers will intervene [34], effectively handle the situation [35], and reward students’ efforts when they report or intervene in bullying [36]. In addition, students’ future reporting behavior is influenced by how teachers respond to students’ initial reporting [37]. Students are less likely to report bullying [33] and rates of bullying are higher in the classroom [38] when students perceive that teachers have low self-efficacy in handling bullying situations. As such, it is important for teachers to demonstrate comfort with and confidence in handling bullying situations. Training teachers to effectively respond to bullying and to encourage and support students who report and intervene in bullying are important components of bullying programs aimed at increasing student bystander intervention.

### Reducing Barriers to Access and Implementation

Successful implementation of bullying prevention programs requires key stakeholder buy-in that can be enhanced by reducing barriers through the reduction of time and cost while increasing training flexibility [39]. We are in the process of translating the in-person STAC intervention into a technology-based format (STAC-T) [40,41] to increase accessibility, particularly for schools in low-income and rural communities. Developing a technology-based teacher module has the potential to enhance program efficacy, reduce implementation barriers [42], and increase program scalability [43]. Although web-based training outcomes (eg, increased knowledge, self-efficacy, and behavioral intentions) are equivalent to in-person training outcomes [42], perceived usefulness [44] and participant buy-in [39] are both important factors that contribute to program acceptability. Therefore, it is important to understand the perspectives of teachers regarding the strengths and barriers related to a technology-based format.

### Initial Development of the STAC Teacher Module

The purpose of the development of the STAC teacher module was to enhance intervention outcomes by equipping teachers with knowledge and skills to appropriately address bullying and support students who witness bullying to intervene as “defenders.” The content of the STAC teacher module was originally developed through focus groups conducted with high school teachers [45] and then adapted for the middle school level through focus groups conducted with middle school personnel, including teachers, an administrator, and a school counselor [46]. Preliminary research with high school teachers indicates that the training was effective in increasing teachers’ knowledge; confidence in supporting student bystanders to intervene as *defenders*; and comfort, confidence, and self-efficacy in intervening in bullying situations [45]. Qualitative data from focus groups conducted with middle school personnel suggest that the content of the STAC teacher module is useful, relevant to, and appropriate for middle school settings [46]. In addition, middle school personnel indicated a need for the program and identified barriers to implementation, including cost, time, and teacher buy-in. Participants also provided feedback on delivering the program in a technology-based format, appreciating the flexibility of a technology-based program while expressing concerns regarding participant engagement.

### Study Objectives

The purpose of this study was to build on our prior research on developing the STAC teacher module, examining the effectiveness of the training, as well as providing data to inform the translation of the training into a technology-based format that can be used as a companion module to the STAC-T program. To date, we have collected posttraining data from high school teachers and qualitative data from middle school teachers and other school personnel. This study represents the next step in the development of the STAC teacher module for middle schools. Specifically, we aimed to evaluate immediate posttraining outcomes (eg, changes in knowledge, confidence, self-efficacy, and teachers’ intention to use the STAC strategies) among middle school teachers and obtain additional feedback from a new group of middle school teachers recruited from a different school district regarding program content, acceptability, need, and delivery via a technology-based format. To achieve these aims, we used a mixed methods research design to answer the following research questions: (1) Do the trained teachers report increases in knowledge and confidence to support students who act as *defenders* and increases in confidence, comfort, and self-efficacy in managing and handling bullying? (2) Do teachers indicate behavioral intentions to use the STAC strategies after training? (3) Is the STAC teacher module acceptable and relevant to middle schools? (4) What was the teachers’ feedback regarding program appropriateness, feasibility, content, perception of need, and desire for web-based training?

## Methods

### Participants

Participants were teachers (N=18) recruited from 1 public middle school in a rural, low-income community in the northwestern United States. We selected the school based on a prior and ongoing research partnership. The school was a Title I school located in a rural community, with 89.1% (304/341) of the student population qualifying for reduced or free lunch. The race or ethnicity of the student body included 64.5% (220/341) Hispanic, 34.3% (117/341) White, and 1.2% (4/341) other. Of the 18 participants who completed the pretest survey, 17 (94%) completed the immediate posttest survey. The final sample comprised 76% (13/17) women and 24% (4/17) men. Among the participants, ages ranged from 25 to 62 (mean 44.76, SD 12.16) years, and years of experience as a middle school teacher ranged from 1 to 26 (mean 10.24, SD 7.82) years. Most of the teachers in the sample were female (13/17, 76%) and identified as White (16/17, 94%). Of the 17 teachers who completed the immediate posttest survey, 6 (35%) signed up and participated in the focus group. The sample comprised 35% (6/17) women who identified as White. A series of chi-square analyses and 2-tailed independent-sample *t* tests revealed no statistically significant demographic differences between those who participated in the focus group and those who did not participate.

### STAC Teacher Module for Middle School

#### Overview

The STAC teacher module is a 50-minute module that includes (1) normative feedback regarding the prevalence of bullying to engage teachers; (2) didactic information about bullying, including the definition and types of bullying and the associated negative consequences; (3) a review of the student STAC strategies and corresponding strategies that teachers can use to support students who act as “defenders,” followed by role-plays to reinforce skill acquisition; and (4) information about “perceptions vs. facts” about bullying that can influence how teachers shift the school climate and demonstrations of how teachers can apply research-based strategies to positively influence and shift the school climate.

#### Normative Feedback

The normative feedback module begins with teachers estimating the local and national prevalence rates related to bullying among middle school students. Example questions include the following: (1) What percentage of middle school students say that they have been bullied in the past year? (2) What percentage of middle school students say that they have been cyberbullied in the past year? (3) Rank the order of the location middle school students are most frequently bullied (ie, hall/stairwell, classroom, cafeteria, outside of school grounds, and bathrooms/locker rooms), and (4) Among middle school students, what percentage of students report bullying to an adult?

After the teachers indicate their estimates, trainers provide actual prevalence data in comparison with their responses and facilitate a brief discussion regarding discrepancies.

#### Bullying Education

Teachers are presented with the definition of bullying, including examples of behaviors that would not be considered bullying (eg, what is not bullying). Trainers also discuss different types of bullying (ie, physical, verbal, relationship, and cyberbullying), negative associated consequences for students who are targets or bystanders, and positive outcomes for students who are trained and intervene as “defenders.” This information is presented in a manner that parallels the STAC training for students.

#### STAC Strategies and Role-plays

##### Overview

Trainers present the 4 STAC strategies (ie, *Stealing the Show*, *Turning it Over*, *Accompanying Others*, and *Coaching Compassion*) that students are taught to use to intervene as *defenders* when they witness bullying at school. Next, trainers discuss the skills that teachers can use to support students using each of the 4 STAC strategies. After each strategy is presented, trainers ask for teacher volunteers to engage in a role-play to practice the skills the teachers can use to support students acting as “defenders.” The 4 STAC strategies with the corresponding teacher strategies are outlined in the following sections.

##### Stealing the Show

Students are taught to use humor or distraction to interrupt a bullying situation. Teachers can support students using this strategy by approaching the situation, joining in the conversation, or laughing at a joke that appropriately interrupts the bullying situation. Teachers are encouraged to disperse the peer group so that the perpetrator or perpetrators do not have access to a peer audience. Teachers are also encouraged to reinforce the *defender* with positive feedback, check in on the target, and report the situation to the administration when appropriate.

##### Turning It Over

Students are taught to use *Turning it Over* or reporting the bullying situation to an adult at school, and they are encouraged to always use this strategy if they witness physical or cyberbullying. Teachers can support students who report bullying by assuring them that they did the right thing. Trainers encourage teachers to reinforce to students that bullying is not acceptable and that it requires maturity and strength to report bullying and ask for help. As students generally believe that their peers will perceive them as a “Snitch” if they report bullying to adults [47], teachers are also encouraged to share with *defenders* that research shows that students are generally supportive of their peers who report bullying. In addition, trainers instruct teachers to share with students that it often requires continued documentation for adults to be able to take significant action and that a process may be occurring outside of a student’s awareness because of issues related to confidentiality. As such, teachers should encourage *defenders* to continue reporting and, in the case of cyberbullying, documenting by taking immediate screenshots or pictures. Teachers should also follow up and report bullying to their administration when appropriate.

##### Accompanying Others

Students are taught to use *Accompanying Others* to befriend or support peers whom they witness being a target of bullying. Student *defenders* can use this strategy without calling attention to the situation by inviting students who were targeted to sit with them at lunch or walk with them to class. Depending on the nature of their relationship, *defenders* can share with the target that they witnessed what happened, state that the perpetrator’s behavior was unacceptable, and ask whether they would like to talk about it. Teachers are encouraged to let students who use this strategy know that they are being a good friend and positively affecting their peer’s experience at school. Trainers also encourage teachers to reinforce to *defenders* that by befriending targets, they are communicating to their peers that they care about them and that they are not alone at school. Furthermore, teachers are instructed to encourage *defenders* to check back in with their peers who were targeted the day following the incident to build an acquaintanceship or friendship, as well as to follow up with the targets themselves or report the bullying incident if appropriate.

##### Coaching Compassion

Students are taught to use *Coaching Compassion* to gently confront students who perpetrate bullying behaviors during or after a bullying incident. Only students who are older, have a higher social status, or are good friends with the perpetrators are encouraged to use this strategy. The goal is to raise the awareness of students who bully that bullying is unacceptable while fostering empathy toward the target. Trainers instruct teachers to support students using this strategy by paying close attention to the situation and becoming involved if necessary to ensure safety. In addition, teachers are encouraged to reinforce to *defenders* that they did the right thing in intervening and that bullying is unacceptable and share that research indicates that for students who bully infrequently, increasing awareness and empathy often decreases bullying [48].

#### Perceptions Versus Facts About Bullying and Teachers’ Role in Shaping the School Climate

Trainers present statements related to bullying and school climate, and teachers are asked whether these statements are true or false. Trainers then discuss the correct answers and supporting research. For example, teachers are asked (1) whether most students have negative attitudes toward peers who report bullying to teachers; (2) whether students perceive that teachers think bullying is developmentally appropriate and that teachers do not care enough about bullying; and (3) in schools where teachers appear to be less judgmental of bullying, whether students are bullied more often. The training concludes with a demonstration of a teacher intervening in a classroom bullying situation reinforcing strategies that research supports are effective in positively shifting the school climate.

### Procedures

Participant recruitment and data collection occurred during the spring of 2022. The inclusion criterion was being a middle school teacher at the participating school. The exclusion criterion was being a staff member other than a teacher. The team worked with the school principal to recruit all teachers from 1 middle school to be trained on the STAC teacher module. The first author (AM) and a Master’s student conducted study recruitment, data collection, and the STAC teacher module training in person at the school during a professional development day. The researchers conducted the informed consent process immediately before collecting the baseline data, which was then followed by the STAC teacher module and postintervention data collection. The pre- and postintervention data collection took 15 to 20 minutes to complete, and the training lasted 50 minutes. After postintervention data collection, the researchers invited all study participants to sign up for a follow-up focus group that occurred within a week of the training and was conducted on the web. A doctoral student and a master’s student conducted the focus group, which lasted approximately 45 minutes and was recorded for verbatim transcription. Participants received a US $50 Amazon gift card as an incentive to participate in the STAC teacher module and complete pre- and posttraining surveys. Participants also received a US $50 Amazon gift card as an incentive to participate in the follow-up focus group.

### Ethics Approval

We used active informed consent for this study. The researchers provided the participants with an informed consent form that contained information about the study, including purpose and background, procedures, risks or discomforts, extent of confidentiality, benefits, payment, and how to contact the primary investigator and the institutional review board of the university. All study procedures were approved by the institutional review board of the university (101-SB21-051) and by the school district. Although the principal recruited teachers to participate in the training, the research team members conducted study recruitment. Teachers were informed that their participation in the study procedures (ie, completing surveys or participating in a focus group) was voluntary and that they could choose not to participate or withdraw from the study at any time without penalty. We believe that this procedure minimized any pressure teachers may have felt to participate in the research because of the principal’s role in recruiting teachers to participate in the training.

### Measures

#### Demographic Survey

The teachers completed a brief demographic questionnaire that included questions about age, gender, race or ethnicity, and years of experience teaching.

#### Knowledge and Confidence to Support Defenders

The Teacher-Advocates Pre and Post Scale [45] was used to measure knowledge and confidence in supporting “defenders.” The questionnaire comprises 11 items that measure knowledge of bullying behaviors, knowledge of how to support students using the STAC strategies, and confidence in supporting students who intervene in bullying situations. Examples of items include “I know what verbal bullying looks like,” “I know how to support students who reach out to students who are targets of bullying,” and “I feel confident in my ability to do something helpful to support students who report bullying to me.” Items are rated on a 4-point Likert scale ranging from 1 (*totally disagree*) to 4 (*totally agree*). All the items are summed to compute the total scale score. Possible scores range from 11 to 44. Higher scores reflect higher levels of knowledge and confidence. The Teacher-Advocates Pre and Post Scale has established internal consistency, with Cronbach α ranging from .72 to .95 [45]. The Cronbach α was .71 for this study.

#### Confidence in Managing Bullying

The Teacher’s Attitudes about Bullying Questionnaire [49] is a 22-item questionnaire that contains 5 subscales. We used the 3-item Confidence in Managing Bullying Subscale, which includes the items “I am confident that I will know what bullying is when I see it,” “I am confident that I will know how to respond if one of my students is being victimized by a peer,” and “I am confident that I will put my knowledge into practice and actively respond in bullying situations.” Items are rated on a 5-point Likert scale ranging from 1 (*strongly disagree*) to 5 (*strongly agree*). All the items are summed to compute the total scale score. Possible scores range from 3 to 15. Higher scores reflect higher levels of confidence in managing bullying. The Cronbach α was .67 for this study.

#### Comfort With Managing Bullying

The teachers’ comfort with managing bullying was measured using items from the National Education Association Bullying Survey [50]. The teachers were asked the question “How comfortable would you feel intervening when you see the following bullying behaviors?” followed by four types of bullying and their definitions: (1) physical (hitting, pushing, or kicking), (2) verbal (general teasing or name calling), (3) relational (rumor spreading or excluding someone from a group), and (4) cyberbullying (sending or posting harmful material or engaging in other forms of social aggression using the internet or other digital devices, such as mobile phones). Items are rated on a 4-point Likert scale ranging from 1 (*very uncomfortable*) to 4 (*very comfortable*). All the items are summed to compute the total scale score. Possible scores range from 4 to 16. Higher scores reflect higher levels of comfort with managing bullying. Items were summed to create the scale. The Cronbach α was .71 for this study.

#### Bullying Self-efficacy

The teachers’ self-efficacy in handling bullying situations was measured using 2 items from the National Education Association Bullying Survey [50]. The items “I have effective strategies for handling bullying” and “I have effective strategies for supporting students to intervene in a bullying situation” are rated on a 4-point Likert scale ranging from 1 (*strongly disagree*) to 4 (*strongly agree*). All the items are summed to create the scale. Possible scores range from 2 to 8. Higher scores reflect higher levels of self-efficacy. The Cronbach α was .81 for this study.

#### Intention to Use Teacher STAC Strategies

The intention to use teacher STAC strategies was measured using an adapted version of the Use of STAC Strategies [28] for students. The items were adapted from the student version to be appropriate for teachers. The 4-item measure asks teachers the following: “How likely are you to support students using these strategies to intervene in bullying in the future?: a) Stealing the Show—support students using humor or distraction to get the attention away from the bullying situation, b) Turning it Over—support a student who reported bullying to you or supporting students to report to a principal or SRO, c) Accompanying Others—support students who reach out to the student who was the target of bullying, or d) Coaching Compassion—support students who help the student who bullied develop empathy for the target.” Items are rated on a 5-point Likert scale ranging from 1 (*very unlikely*) to 5 (*very likely*), with higher scores reflecting higher levels of intention to use the strategies.

#### Acceptability and Relevance of the Teacher STAC Training

Acceptability (ie, ease of use or utility) and relevance of the STAC training were assessed using a social validity survey comprising 8 items. Items were ranked on a 4-point scale from 1 (*strongly disagree*) to 4 (*strongly agree*), with higher scores reflecting higher levels of acceptability and relevance. The survey was based on social validity surveys used to assess the appropriateness of interventions adapted for a new population with demonstrated reliability and validity [51,52]. The Cronbach α was .97 for this study.

#### Interview Questions

Within a week of being trained and completing the posttraining survey, a subset of teachers (6/17, 35%) participated in a focus group. Participants were asked semi–open-ended questions about the relevance and appropriateness of the training. Participants were asked (1) what they liked and did not like about the training; (2) what information was missing from the training to equip teachers to support students to act as *defenders* in a bullying situation; (3) how useful they perceived the training was to prepare teachers to address the problem of bullying at school; (4) whether the content of the program was relevant to and appropriate for their students and community; (5) what was the practicality and workability of the training for their school setting; (6) what types of similar training they had received, including training specifically about bullying; (7) whether they thought there was a need and whether they would use this training at their school; (8) what barriers may prevent their school from adopting or implementing a bullying intervention program; (9) what were the strengths or barriers to implementing this program as a web-based program; and (10) what was the usefulness of web-based training.

### Data Analysis

#### Quantitative

Quantitative data from the questionnaires were analyzed using SPSS (version 28.0; IBM Corp). Before conducting the statistical analyses, the data were examined for outliers and normality, and all variables were within the normal range for skewness and kurtosis. We computed descriptive statistics for all variables at pre- and posttest measurements. We conducted a series of paired-sample *t* tests to evaluate changes from pre- to posttest measurements. All analyses were considered significant at *P*<.05. The Cohen *d* was used to measure effect size, with the magnitude of effects interpreted as follows: small (*d*=0.20), medium (*d*=0.50), and large (*d*=0.80) [53].

#### Qualitative

One team member, who facilitated the focus group, transcribed the data verbatim. The data analysis team comprised 2 faculty members with expertise in qualitative data analysis and a doctoral student. The lead analyst developed an analysis plan. First, analysts wrote an individual precoding memo reflecting on potential biases and assumptions about the research questions. Subsequently, they participated in a preanalysis meeting where they discussed initial memos and coordination for the analysis process. Team members used thematic analysis, which is a phenomenological approach focusing on participants’ experiences, and an inductive approach to coding the transcript and interpreting the data [54,55]. Analysts coded the focus group transcript individually, wrote a postcoding memo, and met twice to achieve a consensus on themes and finalize the results. The team used the participants’ quotes to resolve disagreements. An external auditor reviewed the results and provided the team with feedback through email correspondence. No identifying information was included in the interview transcripts.

## Results

### Quantitative Analysis

#### Posttraining Outcomes

Means, SDs, and statistical contrasts for pre- to posttest training outcomes are presented in Table 1. The teachers reported an increase in knowledge and confidence to support *defenders* (*P*<.001), confidence in intervening in bullying situations (*P*<.001), comfort with intervening in bullying situations (*P*<.001), and bullying self-efficacy (*P*<.001). All the effect sizes were large. The results support the effectiveness of the teacher training in increasing knowledge and confidence in both working with student bystanders and intervening directly in bullying situations from before the training to after the training.

**Table 1:** Means, SDs, and statistical contrasts for paired-sample *t* tests.

Item	Pretest time point, mean (SD)	Posttest time point, mean (SD)			
	Values, mean (SD)	Values, mean (SD)	*t* test (*df*)	*P* value	Cohen *d*
Knowledge and confidence in supporting *defenders*	30.71 (2.95)	37.34 (3.90)	8.31 (16)	<.001	2.02
Confidence in intervening in bullying	10.47 (1.70)	13.12 (1.54)	7.09 (16)	<.001	1.72
Comfort with intervening in bullying	11.42 (1.62)	13.06 (1.95)	4.20 (16)	<.001	1.02
Bullying self-efficacy	4.65 (0.86)	6.82 (1.01)	9.44 (16)	<.001	2.29

#### Intention to Use Teacher STAC Strategies

The teachers’ ratings of their intention to use teacher STAC strategies are reported in Table 2. As seen in Table 2, most teachers indicated that they were likely or very likely to use the STAC strategies to support students who intervene in bullying in the future. For specific strategies, >90% (16/17, 94%) reported that they would be likely or very likely to support students using *Stealing the Show*, >90% (16/17, 94%) reported that they would be likely or very likely to support students using *Turning it Over*, 100% (17/17) reported that they would be likely or very likely to support students using *Accompanying Others*, and >85% (15/17, 88%) reported that they would be likely or very likely to support students using *Coaching Compassion.*

**Table 2:** Intention to support students using STAC strategies in the future (N=17).

Strategy	Agreement, n (%)				
	Very likely	Likely	Not sure	Unlikely	Very unlikely
Stealing the Show	7 (41)	9 (53)	1 (6)	0 (0)	0 (0)
Turning it Over	8 (47)	8 (47)	1 (6)	0 (0)	0 (0)
Accompanying Others	6 (35)	11 (65)	0 (0)	0 (0)	0 (0)
Coaching Compassion	5 (31)	9 (56)	2 (13)	0 (0)	0 (0)

#### Acceptability and Relevance of the Teacher STAC Training

The percentage of agreement for the social validity survey items is reported in Table 3. Overall, the scores suggest a very high level of program acceptability and relevance. Most teachers found the STAC teacher module to be easy to understand (16/17, 94%), useful (16/17, 94%), interesting (15/17, 88%), and relevant (16/17, 94%) to middle school teachers. Most teachers also indicated that they had learned something from the program (16/17, 94%) and would recommend it to other teachers at their school (16/17, 94%).

**Table 3:** Participants reporting agreement with social validity items (N=17).

Item	Agreement, n (%)			
	Strongly agree	Agree	Disagree	Strongly disagree
The STAC teacher training was easy to understand.	11(65)	5 (29)	0 (0)	1 (6)
The STAC teacher training was useful.	11 (65)	5 (29)	0 (0)	1 (6)
The STAC teacher training was interesting.	9 (53)	6 (35)	0 (0)	2 (12)
The STAC teacher training information was relevant to middle school teachers.	14 (82)	1 (6)	1 (6)	1 (6)
The STAC teacher training examples of bullying were relevant to middle school teachers.	11 (65)	5 (29)	0 (0)	1 (6)
The STAC teacher training strategy role-plays were relevant to middle school teachers.	11 (65)	5 (29)	0 (0)	1 (6)
I learned something from the STAC teacher training.	12 (71)	4 (24)	0 (0)	1 (6)
I would recommend the STAC teacher training to other teachers at my school.	13 (77)	3 (18)	0 (0)	1 (6)

### Qualitative Analysis

#### Overview

Qualitative feedback for the STAC teacher module supported the quantitative findings and was positive overall, with participants sharing the perception that the STAC teacher module was useful, relevant, and appropriate, as well as sharing ways of improving the program. In addition, teachers shared positive thoughts about program feasibility, the need for bullying-specific training for teachers, and strengths of and implementation barriers to a web-based program. The results are presented in the following sections, organized according to the following themes: (1) positive program attributes; (2) relevance and appropriateness; (3) program feedback; (4) feasibility, need, and current program offerings; (5) potential barriers; and (6) web-based offering.

#### Positive Program Attributes

Participants spoke positively about the STAC teacher module, including liking the teacher strategies to support students who report bullying, finding the strategies easy to implement, and finding the role-plays useful. Participants also liked that the STAC program provides students with the knowledge and skills to intervene in bullying situations. A participant shared the following:

You know there are so many things to be concerned about in a classroom and a lot of bullying, I think, is pretty under the radar. So, having the tools to know what to look for and maybe how to interpret some of the things we see. I think it’s really beneficial.

When talking about the strategies they had learned, a teacher added the following:

These are just simple easy things, it is not something that is super difficult or a lot of steps you have to remember to be able to implement some of those strategies so that is really helpful.

Another participant indicated the following:

...the role-playing did help. It just lets you know, yeah, it’s okay to say this and you know gives you something to fall back on.

When discussing the value of empowering students, a participant stated the following:

You know just to be able to have those tools, so they [students] can be empowered to help other kids or to help themselves.

#### Relevance and Appropriateness

When asked whether the STAC teacher module was relevant to and appropriate for their school and community, all participants expressed agreement. A teacher stated the following:

I guess if we see it in our schools it’s happening in our community too. If we can deal with it here hopefully that will bleed out into the community and have an effect, there too.

Another teacher indicated the following:

I think there is a need [for the STAC Teacher Module] and I think, you know, there is some staff that are attuned to it. So, I think there would be definitely some teachers that would use it more than others, which is probably good because we need lots of personalities to connect with different student personalities. My guess is that it is really hard to say we are all going to do this and have it really be effective, but I think given the training and the tools there would be more teachers that would implement it.

#### Program Feedback

When participants were asked for feedback regarding areas that were missing from the training, a few participants indicated the need for role-plays with more realistic scenarios; additional guidance to discern whether students were genuinely acting as defenders or being disingenuous, especially when using humor; and concern about the impact of labeling a student as a bully. A participant indicated the following:

Maybe some scenarios of things that actually might occur, so we have a better idea of what to look for because sometimes they look like they are playing and maybe that is okay. But, then other times, they really are not playing, you know, and so what are some, you know, different scenarios that would help us to identify or see some of the things that really affect middle schoolers that we could kind of tune in on.

Another participant added the following:

Yeah, characteristics to look for because it is hard to know what is genuine and what’s not especially when they are in middle school because everything is a joke. Which I like, I use humor as a tool all of the time, but everything is a joke and sometimes a kid will interpret someone’s humor as bullying.

In terms of concerns about labeling students, a teacher stated the following:

The issue that I struggle with is labeling somebody as a bully because once us, as teachers, identify that behavior as bullying, then all of the sudden other students look at that student differently. When it could be that they just needed to learn. I don’t know I struggle with that as well because once you label them as a bully then it is kind of stuck with them and other students look at them differently. And then all of the sudden the bullying actually shifts and they start to look at that kid negatively.

However, another participant responded as follows:

On the other hand sometimes we don’t acknowledge when somebody is a bully. And we just keep justifying their behavior and it makes the kids that are being bullied like victimized more and so I think it is important that we get that conversation going...

#### Feasibility, Need, and Current Offerings

Participants indicated agreement on the program being practical and workable. Participants also spoke about the need for training and support in addition to the current offerings on social-emotional learning and identifying a need for bullying-specific training for teachers. When asked if the program seemed practical and workable, a participant indicated the following: “Yeah, I think that would be great.” Another participant added that “I think it would be a feasible thing to do...” In terms of current offerings, a participant stated the following:

As a district we also have a 21st Century program that serves our elementary school and our middle school kids and they have a huge focus on the social emotional learning.

When asked about training that teachers received specifically about bullying, a participant indicated the following: “Uh, no not enough...We have a little, you know.”

#### Potential Barriers

Participants spoke about concerns regarding cost, potential pushback from parents, and maintaining an open mind. For example, a teacher stated that “Cost, if there is a cost to it—there is going to be one [barrier] there.” Another teacher shared the following:

Yeah, and I think even some kind of pushback from parents, you know, because we’ve seen that kids that you would think are perfect kids, they are the actual ones that are bullying, and their parents can’t believe it.

Finally, a teacher shared the following:

It’s hard to redefine people that you think you know. So, if you think of them as a perfect student and then you hear that they’re a bully as well, well is that really true or is it you know so having that open-mindedness to just say oh I guess what I thought was wrong.

#### Web-Based Programs

When addressing completing the STAC teacher module as a web-based program, participants talked about strengths such as ease of implementation and time efficiency, as well as challenges, including less engagement and missing the in-person connection. Participants also spoke about inherent issues related to technology and the importance of administration buy-in. For example, when speaking about strengths, participants indicated the following:

...you can go back and review it. So, if I’m thinking, okay, what did we say about that, or whatever or the kids need a refresher or those kinds of things. Technology does give us access to that as well as consistency.

Another participant added the following:

Technology would make it really easy to do a follow up with this kind of course...a refresher using technology and a quick setting.

A third participant added the following:

One thing is nice with web-based because you don’t have to have a sub and you don’t have to miss class and that’s really valuable because we spend a lot of time doing sub plans and different things.

However, this participant went on to say the following:

But it’s just not as engaging

to complete the program online

Another participant added the following:

When you’re trying to implement bullying strategies, how to deal and handle and cope, there is something to be said with the interpersonal relationship having someone in person teaching it versus online...

Another teacher stated the following:

...sometimes that removed, that technology piece, you don’t engage the same way as if you’re in person...

Participants also mentioned technology issues as barriers; for example, a teacher stated that “Access to technology, yep.” Finally, a participant shared the following about the importance of buy-in:

Yeah, I think there is buy-in and if there is buy-in from our administration then it’s a go. You know, and I think you kind of need a little bit of our administration to push it out and teachers that really see a need for it to buy-it and to push it out to the other teachers. I think a lot of us do different things as situations arise, we talk about it in our classrooms, that’s—we don’t have anything like whole school

programming

## Discussion

### Principal Findings

The purpose of this study was to build on our prior research on developing the STAC teacher module, examining the effectiveness and acceptability of the training, as well as providing data to inform the translation of the training into a technology-based format that can be used as a companion model to the STAC-T student bystander bullying intervention. Quantitative findings indicated that teachers reported increases in knowledge and confidence to support students who intervene as *defenders* and to directly intervene in bullying situations from before training to after training. The teachers also reported behavioral intentions to use the STAC teacher strategies. Furthermore, the participants’ responses demonstrated high levels of program acceptability and relevance to middle school teachers. Qualitative findings were consistent with the quantitative results and revealed the following themes: (1) positive program attributes; (2) relevance and appropriateness; (3) program feedback; (4) feasibility, need, and current program offerings; (5) potential barriers; and (6) web-based offering.

### Comparison With Prior Work

Consistent with prior research conducted with high school teachers trained in the STAC teacher module [45], quantitative data demonstrated increases in knowledge and confidence to support *defenders* as well as increases in confidence, comfort, and self-efficacy in managing and handling bullying from before training to after training. In addition, the teachers reported that they would use all 4 strategies to support students who act as *defenders* on behalf of targets. Qualitative data supported these findings, indicating that the teachers liked the strategies and found them easy to implement. These findings are important as increases in teacher knowledge and bullying self-efficacy have been associated with directly intervening in bullying [33], which, in turn, could lead to students being more likely to report bullying behavior [34]. By rewarding students’ efforts when they report or intervene in bullying [36], teachers are also likely to reinforce and increase bystander intervention, which has been shown to decrease bullying behaviors [48].

In terms of program acceptability and relevance, quantitative data indicated that most participants (16/17, 94%) reported that the STAC teacher module was easy to understand, useful, and relevant to middle school teachers. Furthermore, most participants (16/17, 94%) indicated that they learned something from the training and would recommend it to others. Consistent with our prior research with middle school personnel [46], the teachers reported that the training was useful, relevant to, and appropriate for middle school settings. Qualitative data supported these findings, with all teachers indicating agreement about the training being relevant. As program adoption and implementation are associated with acceptability and relevance [56], these findings are particularly promising.

The teachers also provided feedback on program feasibility and perception of program need. Although they confirmed the need for bullying programs that equip teachers to intervene and to support students who act as “defenders,” the teachers also spoke about barriers, including cost, as well as the importance of considering parents in bullying programs. These findings are consistent with our previous studies concerning the implementation of both the STAC teacher module [46] and the student STAC-T program [40,41], suggesting that there is a need for a cost-effective STAC teacher module that incorporates parental participation.

Regarding program content, the teachers provided feedback indicating that the role-plays corresponding to the 4 STAC teacher strategies were helpful; however, a few participants expressed a desire for more realistic scenarios to further improve the training. In addition, although a few teachers stated concern about the impact of labeling a student as a bully, they also acknowledged the importance of recognizing bullying behavior. This reluctance to acknowledge bullying is similar to findings from previous studies in which teachers indicated a desire to remain neutral [45]. However, it is important for teachers to be willing to communicate that bullying behaviors are unacceptable, reinforce students who intervene as *defenders* [36], and discipline students who perpetrate bullying [37] to establish a school climate that does not condone or promote bullying.

Finally, when asked about their perspectives on web-based programs, the teachers pointed out strengths, such as ease of implementation and time efficiency, as well as challenges, including less engagement and missing the in-person connection. Participants also suggested that these barriers could be mitigated by administrative buy-in. These findings are similar to prior research conducted with school personnel emphasizing the importance of flexibility while expressing concerns about participant engagement [46]. As training acceptability is related to stakeholder buy-in [39], it may be important to emphasize that web-based training decreases program costs and allows for training schedule flexibility [42]. Designing the training to include web-based assessment and personalized feedback components to individualize the user experience [57] can be integrated to maintain engagement.

### Limitations

Although this study contributes to the literature, certain limitations must be considered. First, we only collected data from teachers in 1 rural, low-income middle school in the northwestern United States. We chose to use only 1 school as this study represents one of a series of studies conducted as formative research on the development of the STAC teacher module. Our prior research included teachers from 2 middle schools. This sample represents teachers from a third middle school.

Furthermore, because of the small sample size and lack of a control group for the quantitative portion of the study, we cannot make causal attributions or generalize our findings to the larger middle school teacher population. Therefore, it would be helpful for future studies to include middle schools from different regions in the country with greater racial or ethnic diversity and to conduct a randomized trial to assess training efficacy. In addition, as most of the sample was female, the interpretation of the results for men should be made cautiously. Formative research conducted during the development of the prototype application of the STAC teacher module should use purposeful sampling to ensure that men are included. Finally, our findings were based on self-reported data. It is possible that the teachers’ responses to the survey questions and the focus group interview were influenced by their desire to please the researchers. They may have been particularly influenced as team members who trained the teachers were present during both quantitative and qualitative data collection. Future research in which team members who act as trainers are different from those who conduct data collection would be helpful in reducing participants’ potential desirability effects.

### Implications

This study has important implications for the development of the STAC teacher module and the translation of the training into a technology-based format that can be used as a companion module to the STAC-T program. First, the teachers reported increased immediate posttraining outcomes (ie, knowledge, confidence, comfort, and self-efficacy) in managing and handling bullying and supporting student bystanders to intervene in bullying situations. Furthermore, the teachers indicated behavioral intentions to use the STAC strategies after the training and reported that the training was feasible as long as the program was cost-effective. In addition, although the response to the training was very positive, a few teachers indicated a desire for more realistic scenarios for role-plays; as such, it may be beneficial for researchers to conduct additional focus groups with both teachers and students to further investigate student reporting behavior and teachers’ responses. Furthermore, participants identified potential parent pushback as a barrier. Therefore, the development of a parent training intervention to educate parents about the STAC program by providing information that parallels the STAC teacher module may be an important next step in program development.

The findings of this study also support the initial prototype development and testing of a technology-based version of the STAC teacher module. The teachers pointed out that the benefits associated with web-based training included ease of implementation and time efficiency. However, the teachers cautioned that maintaining engagement in web-based training is important. The teachers talked about the importance of administrator buy-in for successful adoption and implementation. Thus, when designing an internet-based program to maximize teacher engagement, integrating feedback from both teachers and administrators will be an essential element in developing a technology-based version of the STAC teacher module.

### Conclusions

Bullying is a significant problem for youth in the United States, reaching its peak in middle school. Although training students in the STAC program to act as *defenders* is a promising approach to bullying interventions, equipping teachers to support students who intervene may increase the effectiveness of the program. Findings from this study demonstrate immediate posttraining outcomes, including increased knowledge, confidence, comfort, and self-efficacy, as well as teachers’ behavioral intentions to use the STAC strategies. Furthermore, this study demonstrates program acceptability, relevance, feasibility, and the need for a teacher training intervention, as well as interest in a web-based translation. This study provides support for the effectiveness of the STAC teacher module and provides data to inform the translation of the training into a technology-based format that can be used as a companion model to the STAC-T bystander bullying intervention.
